# Risk Factors for Neck and Upper Extremity Disorders among Computers Users and the Effect of Interventions: An Overview of Systematic Reviews

**DOI:** 10.1371/journal.pone.0019691

**Published:** 2011-05-12

**Authors:** Johan H. Andersen, Nils Fallentin, Jane F. Thomsen, Sigurd Mikkelsen

**Affiliations:** 1 Danish Ramazzini Centre, Department of Occupational Medicine, Regional Hospital Herning, Herning, Denmark,; 2 Center for Physical Ergonomics, Liberty Mutual Research Institute for Safety, Hopkinton, Massachusetts, United States of America; 3 Department of Occupational and Environmental Medicine, Copenhagen University Hospital Bispebjerg, Copenhagen, Denmark; Marienhospital Herne - University of Bochum, Germany

## Abstract

**Background:**

To summarize systematic reviews that 1) assessed the evidence for causal relationships between computer work and the occurrence of carpal tunnel syndrome (CTS) or upper extremity musculoskeletal disorders (UEMSDs), or 2) reported on intervention studies among computer users/or office workers.

**Methodology/Principal Findings:**

PubMed, Embase, CINAHL and Web of Science were searched for reviews published between 1999 and 2010. Additional publications were provided by content area experts. The primary author extracted all data using a purpose-built form, while two of the authors evaluated the quality of the reviews using recommended standard criteria from AMSTAR; disagreements were resolved by discussion. The quality of evidence syntheses in the included reviews was assessed qualitatively for each outcome and for the interventions.

Altogether, 1,349 review titles were identified, 47 reviews were retrieved for full text relevance assessment, and 17 reviews were finally included as being relevant and of sufficient quality. The degrees of focus and rigorousness of these 17 reviews were highly variable. Three reviews on risk factors for carpal tunnel syndrome were rated moderate to high quality, 8 reviews on risk factors for UEMSDs ranged from low to moderate/high quality, and 6 reviews on intervention studies were of moderate to high quality. The quality of the evidence for computer use as a risk factor for CTS was insufficient, while the evidence for computer use and UEMSDs was moderate regarding pain complaints and limited for specific musculoskeletal disorders. From the reviews on intervention studies no strong evidence based recommendations could be given.

**Conclusions/Significance:**

Computer use is associated with pain complaints, but it is still not very clear if this association is causal. The evidence for specific disorders or diseases is limited. No effective interventions have yet been documented.

## Introduction

Upper extremity musculoskeletal disorders of upper limb (UEMSDs) and carpal tunnel syndrome (CTS) have been linked to keyboard and Visual Display Unit (VDU) use since the beginning of the seventies [1]–[7].

The majority of UEMSDs are characterized by recurrent episodes of pain and accompanied by disability, varying in severity and impact. Most of the episodes are self-limiting and subside within days or weeks, while some end up with long-lasting chronic problems. Risk factors from physical, psychological, and social domains have been identified, but the relative contribution of the various risk factors to the onset and aggravation of UEMSDs is not clear. As a result, controversies still exist regarding the degree of work-relatedness of UEMSDs [8], [9].

In two reviews addressing musculoskeletal disorders among computer users in the Occupational Medicine State of the Art Reviews in 1999, the conclusions were not that cautious [10], [11], and included statements such as ‘computer-related risk factors demonstrating a consistent relationship with MSDs include [1] computer use with sustained awkward postures, [2] long duration of computer use, and [3] work organization factors’ [11], and ‘upper extremity MSDs are exposure-related in men and women using VDUs, and there is adequate scientific knowledge regarding specific aspects of VDU work to prevent many of the MSDs.’ [10]. With one exception, these conclusions were made exclusively on the basis of cross-sectional studies [12]. Since the anecdotic stories in the seventies, the last decades have been characterized by a steady increase in the number of published studies on computer work and UEMSDs. The studies generally fall into one of two categories: either experimental studies trying to identify a possible pathophysiology of computer related disorders [13]–[17], or intervention studies and epidemiological studies focusing on the association between workplace risk factors and musculoskeletal outcomes.

The pathophysiological or mechanistic studies are not included in this overview. Instead the scope has been to summarize the knowledge and synthesize the evidence gained from the large number of risk factor studies, including prospective and intervention studies, which have been published since the 1999 reviews [10], [11]. Although several systematic reviews on computer work and UEMSDs have been published in recent years in an attempt to provide this kind of information the conclusions in the reviews are often in discord and the heterogeneity has created a situation of confusion rather than of clarity.

The specific scope of this paper is thus to provide an overview of all systematic reviews on risk factors and intervention studies published since the before mentioned first reviews in 1999 and covering vocational computer use and one of the following outcomes 1) Carpal tunnel syndrome, or 2) UEMSDs, including specific diagnoses as well as nonspecific musculoskeletal disorders and complaints from the neck and upper extremity.

The aim has been to provide a synthesis of the evidence on computer work and the risk of carpal tunnel syndrome and UEMSDs and the effect of workplace interventions. The synthesis was based on a thorough evaluation of the quality of the reviews included and an assessment of the conclusions regarding the association between computer work and UEMSDs and recommendations for interventions.

## Methods

### Criteria for considering reviews for inclusion

The criteria for considering reviews for inclusion in the overview were derived from their stated objectives. A review to be considered systematic should as a minimum report search methods, inclusion criteria and at least one or more aspects of validity assessment of original studies. We included a few reviews not fulfilling all these criteria in order to assess the difference between reviews based on the level of rigorousness in their reporting, and to have a more complete list of reviews of computer work and CTS and UEMSDs.

### Search methods for identification of reviews

Based on initial experiments a PubMed search profile was developed:

“musculoskeletal”[All Fields] OR “carpal tunnel syndrome”[All Fields] OR “hand”[All Fields] OR “upper limb”[All Fields] OR “wrist”[All Fields] OR “neck”[All Fields] OR “forearm”[All Fields] OR “elbow”[All Fields] OR “shoulder”[All Fields] **AND** “computers”[All Fields] OR “computer terminals”[All Fields] OR “vdt”[All Fields] OR “keyboards”[All Fields] OR “office”[All Fields] OR “computer”[All Fields] OR “keyboard”[All Fields] OR “vdu”[All Fields]) OR “Computers”[Mesh] OR “Computer Terminals”[Mesh] **AND** limits: “humans”[Mesh Terms] AND Review[ptyp] AND “1999/01/01”[PDAT]: “2010/05/04”[PDAT]).

This profile was run first and later translated to Embase, CINAHL and Web of Science. Additional references on papers and other commissioned reviews were provided through contacts to the authors of the included reviews.

### Data collection and analysis

#### Selection of relevant reviews

The primary author applied the selection criteria to the list of potentially relevant reviews. The selection process was accomplished in three steps, first based on only the titles, second on the abstracts, and finally on the retrieved full papers. In case of doubt, a decision on inclusion or not was obtained by discussion and argued agreement with one of the other authors (NF).

#### Data extraction and management

Information on study designs included the number of original studies in the review, the number of studies concerning a specific topic, and the conclusions reached by the review authors. The references to original studies included in the reviews were obtained and the degree of overlap between the different reviews regarding included studies was recorded.

#### Critical appraisal

Review articles were critically appraised by two authors (JHA, NF) using AMSTAR Assessment of Multiple Systematic Reviews, an 11-item tool designed to appraise the methodological quality of systematic reviews [18], [19]. The AMSTAR items are scored as ‘Yes’, ‘No’, ‘Can't answer’ or ‘Not applicable’. Disagreements on item scores were resolved by discussion.

The AMSTAR criteria comprise:

1. ‘a priori’ design provided;
2. duplicate study selection/data extraction;
3. comprehensive literature search;
4. status of publication as inclusion criteria;
5. list of studies included/excluded provided;
6. characteristics of included studies documented;
7. scientific quality assessed and documented;
8. appropriate formulation of conclusions;
9. appropriate methods of combining studies;
10. assessment of publication bias; and
11. conflict of interest statement.

The maximum score on AMSTAR is 11, and 0–4 indicate that the review is of low quality, 5–8, of moderate quality, and 9–11, of high quality.

We considered conflict of interest statement as of minor importance in this topic of computer use and musculoskeletal health. Therefore, our rating differed from the original AMSTAR and with a maximum obtainable score of 9 we considered 0–4 as low quality, and 5 or more as moderate to high quality.

#### Quality assessment and synthesis of the evidence

Due to the heterogeneity of studies we did not adopt a formal grading of the synthesized evidence, e.g. like the GRADE approach [20]. We made a more qualitative assessment of (1) the overall quality of the presented evidence, (2) the overall likelihood that the observed associations were due to a causal relationship, and (3) the existence of evidence based recommendations for practice derived from intervention studies.

### Ethics

No ethical approval was required.

## Results

### Selection of reviews

**Figure 1:**
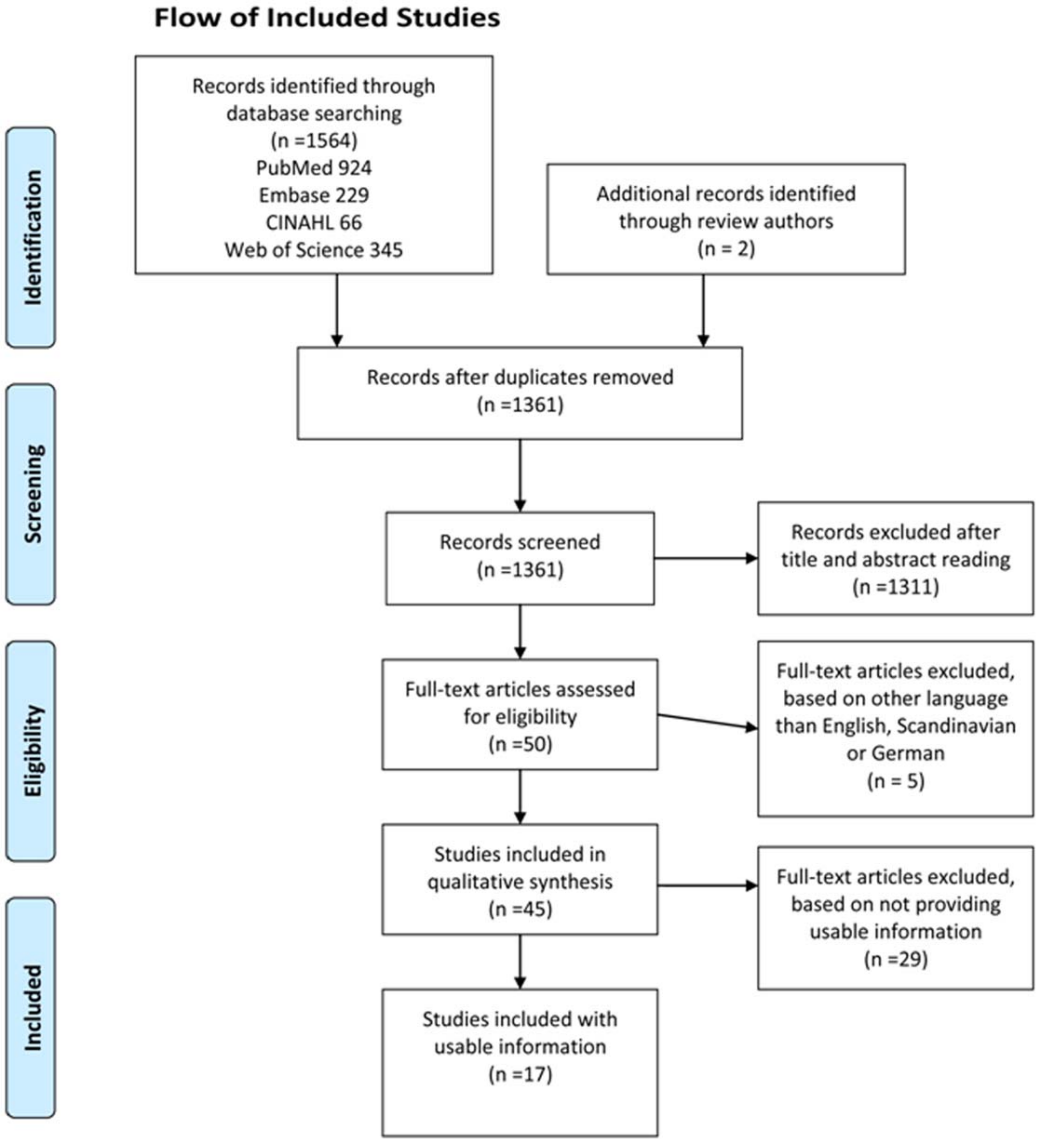
Flow of Included Studies.

The flow diagram shows our selection of reviews identified by the electronic searches and supplied with reviews provided by the authors of the reviews Figure 1. Appendix B presents a list of the reviews that were excluded based on full text assessments along with an explanation. In total 17 relevant systematic reviews were included [12], [21], [36]. Three reviews addressed the specific outcome carpal tunnel syndrome [21], [26], [27]; seven reviews covered UEMSDs more broadly [22], [25], [31], [32], while one review was examining risk factors for neck pain in the working population [28]. Six reviews covered intervention studies [29], [30], [33]–[36].

The three carpal tunnel reviews included a total of 11 primary studies, and the two latest reviews were with few exceptions based on the same original studies. The first published review [Palmer et al.] only included studies from before 1 January 2005 explaining the lack of overlap for at least 3 of the original studies.

The seven reviews on UEMSDs covered in total 80 original studies, but the number of studies per review varied from 9 to 44. This marked difference could partly be explained by difference in study purpose and inclusion criteria. The review by IJmker [31] included only prospective studies, and eight of the nine prospective studies were also included in the most comprehensive review [23]. The recent review by Waersted et al. was restricted by requiring clinical assessments of UEMSDs for their included studies, while at the same time including far more intervention studies for their assessment of causality than the other reviews [32].

The six reviews of intervention studies differed in their target population focusing specifically at computer users [34] and office workers [36] or more broadly on interventions on neck and upper extremity MSDs among a variety of occupations, including office workers [29], [30], [33], [35]. We extracted only the part of the reviews looking specifically at computer users or office workers and dealing with musculoskeletal complaints or disorders from neck and upper extremity. In total, the six reviews included 53 original papers of interventions, varying from 6 [29] to 42 [35]. The study with only 6 papers has recently been withdrawn from the Cochrane database, effective from Issue 3, 2009, because it is considered out-of-date.

**Figure 2:**
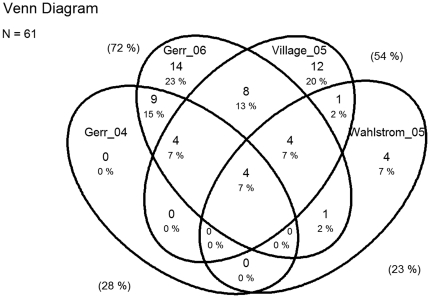
Overlap of original papers included in reviews from 2004 to 2006 of risk factors for Upper Extremity Musculoskeletal Disorders (UEMSDs). Numbers and percentages inside the ellipses show the overlap of the original studies included in the four reviews. Percentages outside the ellipses illustrate the percentage of all 61 original studies included in each of the four reviews [14], [22], [23], [24].

**Figure 3:**
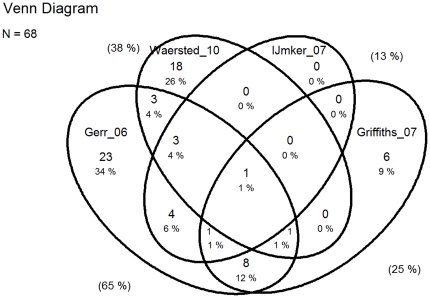
Overlap of original papers included in reviews from 2006 to 2010 of risk factors for Upper Extremity Musculoskeletal Disorders (UEMSDs). Numbers and percentages inside the ellipses show the overlap of the original studies included in the four reviews. Percentages outside the ellipses illustrate the percentage of all 68 original studies included in each of the four reviews [23], [25], [31], [32].

**Figure 4:**
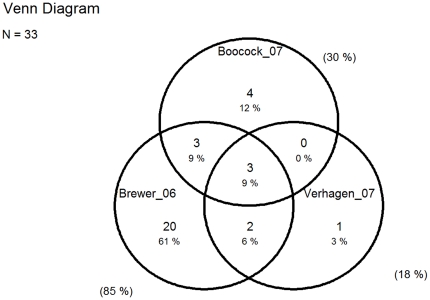
Overlap of original papers on intervention studies among office workers included in reviews from 2006 to 2007. Numbers and percentages inside the circles show the overlap of the original studies included in the four reviews. Percentages outside the circles illustrate the percentage of all 33 original studies included in each of the four reviews [29], [33], [34].

**Figure 5:**
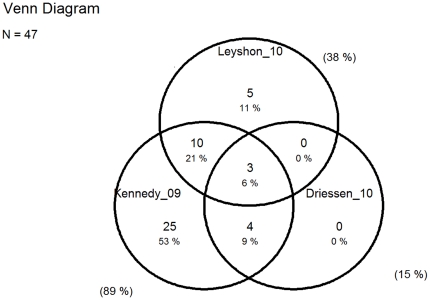
Overlap of original papers on intervention studies among office workers included in reviews from 2009 to 2010. Numbers and percentages inside the circles show the overlap of the original studies included in the four reviews. Percentages outside the circles illustrate the percentage of all 47 original studies included in each of the four reviews [30], [35], [36].

The degrees of overlap of original papers between reviews are presented in Venn diagrams (Figure 2, Figure 3, Figure 4, and Figure 5).

### Quality of original studies included in reviews

#### Carpal tunnel syndrome

One of the three reviews used a quality assessment list [26], while the two others used a qualitative approach to discuss study design, exposure assessment and outcome definition. Of the total 11 studies in the reviews four were prospective in design, one was a case-referent study, one was cross-sectional with a case-referent approach, and four were cross-sectional.

#### UEMSDs

The two latest reviews used an identical quality assessment list, originally derived from Dutch studies [49]–[51]. In the Norwegian review relatively small intervention studies had the same or higher quality assessment than large epidemiological studies. The two reviews [14], [25] with low quality score by AMSTAR did not include any stated quality assessment of the included studies. The review by Gerr et al. from 2006 [23] benefits from building on a 2004 review from the same group, including the same 17 studies as in 2004 [24], and adding 27 more.

#### Intervention studies

The quality assessment in the intervention reviews [34]–[36] was based on explicit and comparable criteria, but Leyshon [36] rated the quality scoring in a different way. Verhagen et al. [29] used a Dephi list adopted from [52] to assess quality; Boocock et al. [33] used the modified Cochrane Musculoskeletal Injuries Group scoring system [53], while Driessen used a modified version of this system and an assessment of the overall quality of the evidence based on the GRADE approach [54]. The different approaches produced different results. As an example two studies on arm support [47], [48], were scored as studies of high quality in two reviews [34], [35], of medium quality in one review [36], and of low quality in another [30].

### Synthesis of evidence

#### Carpal tunnel syndrome

The three included reviews focusing specifically on carpal tunnel syndrome consistently concluded that epidemiological evidence for computer use and the occurrence of CTS is insufficient. In contrast, one of the more general reviews on risk factors [22] concluded that the risk for CTS is increased with use of a computer; especially with mouse use for more than 20 hours per week. This conclusion was, however, based on only one original study [37]. This review was also included among the carpal tunnel reviews, but since its conclusion was based on the evidence from a single study the reviews on CTS considered this evidence as insufficient to alter the overall conclusion.

At the same time, the quality assessment of the three CTS reviews in general yielded higher scores than the broad reviews of UEMSDs (Table 1).

**Table 1:** Appraisal of included reviews on risk factors for carpal tunnel syndrome CTS, upper extremity musculoskeletal disorders (UEMSDs) and review of intervention studies among computer users and/or office workers.

	AMSTAR Criteria*											
Author	1	2	3	4	5	6	7	8	9	10	11	Total yes
*UEMSDs*												
Gerr F,2004	Y	N	N	N	N	Y	Y	Y	Y	N	-	5
Wahlström J,2004	N	N	N	N	N	N	N	N	N	N	-	0
Village, J, 2005	Y	N	Y	N	N	Y	Y	N	N	N	-	4
Gerr F, 2006	Y	N	Y	N	N	Y	Y	Y	Y	N	-	6
Griffiths KL, 2007	Y	N	N	N	N	Y	N	N	N	N	-	2
IJmker S,2007	Y	Y	Y	N	N	Y	Y	Y	Y	N	-	7
Waersted M,2010	Y	Y	Y	N	N	Y	Y	Y	Y	N	-	6
*Carpal tunnel syndrome*												
Palmer KT,2007	Y	Y	Y	Y	N	Y	N	Y	Y	N	-	7
Thomsen JF, 2008	Y	N	Y	N	N	Y	Y	Y	Y	N	-	6
van Rijn RM,2009	Y	Y	Y	N	N	Y	Y	Y	Y	N	-	7
*Neck pain*												
Cote P, 2008	Y	N	N	N	N	Y	Y	Y	Y	N	-	5
*Intervention reviews*												
Brewer, 2006	Y	Y	Y	N	N	Y	Y	Y	Y	N	-	7
Verhagen, 2007	Y	Y	Y	N	N	Y	Y	Y	Y	N	-	7
Boocock, 2007	Y	N	Y	N	N	Y	Y	Y	Y	N	-	6
Kennedy, 2009	Y	Y	Y	N	Y	Y	Y	Y	Y	N	-	8
Leyshon, 2010	Y	N	Y	N	N	Y	Y	Y	Y	N	-	6
Driessen, 2010	Y	Y	Y	N	N	Y	Y	Y	N	N	-	6

* The maximum score on AMSTAR is 11, and 0–4 indicate that the review is of low quality, ≥5 of moderate to high quality.

Y = yes, N = no.

#### UEMSDs

The seven reviews had remarkably different conclusions Table 2, ranging from ‘consistent evidence’ [22], ‘extensively researched and generally well established’ [25], to ‘moderate evidence’, and ‘limited evidence’ in the two latest reviews [31], [32]. As previously mentioned the reviews are to a large extent based on different original studies, which is clearly visible in the Venn diagrams (Figure 2 and Figure 3). However, in the majority of the reviews the core body of evidence seemed to be mainly drawn from the same studies. The exception being the two reviews [14], [25] that received the lowest quality score of the in the AMSTAR rating (Table 1).

**Table 2:** The aim and main conclusions from the 11 included reviews on risk factors for carpal tunnel syndrome, UEMSDs, and neck pain among computer users.

	Carpal tunnel syndrome (CTS)
Palmer,	Aim: To assess occupational risk factors for CTS
2007	*Conclusion: The balance of evidence on keyboard and computer work does not indicate an important association with CTS*
Thomsen,	Aim: To examine evidence for an association between computer work and CTS
2008	*Conclusion: There is insufficient epidemiological evidence that computer work causes CTS.*
Van Rijn, 2009	Aim: A quantitative assessment of exposure-response relationships between work-related physical and psychosocial factors and the occurrence of CTS in occupational populations
	*Conclusion: The contradictory findings for computer use and the development of CTS are in agreement with the conclusion of a recent review (Thomsen, 2008).*
	Upper extremity musculoskeletal disorders (UEMSDs)
Gerr, 2004	Aim: The epidemiological evidence examining associations between UEMSDs and computer use posture and keyboard use intensity (hours of computer use per day or per week).
	*Conclusion: Daily or weekly hours of computer use is more consistently associated with hand and arm MSDs than with neck and shoulder MSDs.*
Wahlstrom 2005	Aim: To give a summary of the knowledge regarding ergonomics, musculoskeletal disorders and computer work and to present a model that could be used in future research.
	*Conclusion: None. It is hypothesized that perceived muscular tension is an early sign of musculoskeletal disorder, which arises as a result of work organizational and psychosocial factors as well as from physical load and individual factors.*
Village 2005	Aim: To evaluate the evidence supporting a causal relationship between computer work and musculoskeletal symptoms and disorders (MSDs) of the hand, wrist, forearm, and elbow.
	*Conclusion: There is consistent evidence of a positive relationship across numerous prospective and cross-sectional studies with increased risk most pronounced beyond 20 hours/week of computer use or with increasing years of computer work. The disorders confirmed with physical examinations are wrist tendonitis and tenosynovitis, medial and lateral epicondylitis, and DeQuervain's tenosynovitis. The risk of carpal tunnel syndrome is increased with a use of a computer, especially with mouse use for more than 20 hours per week.*
Gerr 2006	Aim: The epidemiological evidence of associations between upper extremity musculoskeletal symptoms and disorders and keyboard use intensity (hours of computer use-per day or per-week) and computer use postures was explored.
	*Conclusion: A somewhat consistent finding is an observed association between hours of computer use and adverse hand/arm MSD outcomes and, to a slightly lesser extent, between hours of computer use and adverse neck/shoulder outcomes. The conclusion also points to severe methodological limitations in the literature.*
Griffiths 2007	Aim: To draw attention to the potential risks to musculoskeletal health with the computerization of work amongst professional occupational groups.
	*Conclusion: The risk factors for work related musculoskeletal symptoms with computer work have been extensively researched and are generally well established*.
IJmker 2007	Aim: To get a more conclusive insight into the relationship between the duration of computer use and the incidence of hand-arm and neck-shoulder symptoms and disorders, a systematic review of longitudinal studies was performed.
	*Conclusion: This review showed moderate evidence for an association between the duration of mouse use and the incidence of hand-arm symptoms. Indications for a dose-response were found. In addition, the neck-shoulder region seemed less susceptible to exposure to computer use than the hand-arm region.*
Waersted 2010	Aim: To examine the evidence between computer work and neck and upper extremity disorders (except carpal tunnel syndrome).
	*Conclusion: There is limited epidemiological evidence for an association between aspects of computer work and some of the clinical diagnoses. None of the evidence was considered as moderate or strong and there is a need for more and better documentation.*
	Neck pain
Cote	Aim: To identify risk factors for neck pain in workers
2009	*Conclusion:. The Neck Pain Task Force found evidence that workplace physical exposures (i.e., sedentary work position, repetitive work, precision work, awkward work postures, physical work environment, computer workstation setup) and psychosocial exposures (i.e., quantitative job demands and social support at work) are risk factors for neck pain in workers. However, their effects are small and nonspecific; a single one of these exposures is unlikely to cause neck pain on its own. Neck pain has a multifactorial etiology and its development is dependent on the presence of more than one risk factor. The role of working with hands above the shoulders, heavy physical work and computer screen height as risk factors remains unclear*

The most comprehensive review by Gerr et al. [23] concludes that there is a somewhat consistent finding of an observed association between hours of computer use and adverse hand/arm outcomes, and to a slightly lesser extent for neck/shoulder outcomes, but several methodological limitations are addressed and discussed. The review by Waersted et al. was commissioned by the Danish National board of Industrial Injuries, thus focusing on studies with clinical examinations, and they found only limited evidence for an association between computer use and specified disorders of the neck, shoulder, elbow, forearm, or wrist [29].

The reviews in this category cover a time span of six years and there was a tendency for the most recent reviews to have higher AMSTAR score. Individual studies were included in the 7 reviews a mean of 2.04 times [SD = 1.30], but a number of core studies seemed to be common for most reviews. One study [38] was included in six of the reviews, while the papers from NUDATA [39], [40], [44] were included in five of the seven reviews. Nearly half of the original studies - 37 out of 80 - were only included in one of the reviews.

In a synthesis of the evidence presented in the seven reviews the picture is not straight forward.

There seems to be evidence in the reviews for an association between computer use and pain reporting and discomfort, especially from the distal arm and hand.

However when considering prospective studies and accepting the inclusion of clinical criteria incorporated in some of the reviews, the evidence seems moderate or even limited for an association between computer use and UEMSDs.

The one specific review on neck pain [28] was not aimed at computer use, and included only cohort studies. However, 5 studies from the total pool of studies among computer users [38], [41], [44]–[46], were included in the review and contributed to the conclusion that a wide range of workplace physical factors were risk factors for neck pain (Table 2). The multifactorial nature of neck pain was however emphasized as their main conclusion.

#### Intervention studies

The six reviews are fairly consistent in concluding that there is limited evidence for specific interventions and a conspicuous lack of more high quality studies (Table 3). The most comprehensive reviews finds moderate evidence for no effects of workstation adjustments and rest breaks [34], [25], whereas some evidence supported the effect of arm support, based on 2 studies [47], [48]. The reviews had poor overlap as illustrated by the Venn diagrams in Figure 4 and Figure 5.

**Table 3:** The aim and main conclusions from the six included reviews on intervention studies among computer users and/or office workers.

Brewer, 2006	Aim: To identify studies that evaluated the effects work place intervention on visual or upper body musculoskeletal symptoms or disorders among computer users.
	*Conclusion: Moderate evidence was observed for: (1) no effect of workstation adjustment, (2) no effect of rest breaks and exercise and (3) positive effect of alternative pointing devices. Few high quality studies were found that examined the effects of interventions in the office on musculoskeletal health.*
	Aim: To determine the effects of conservative interventions for work-related “complaints of the arm, neck and/or shoulder” (CANS) in adults.
Verhagen, 2007	*Conclusion: There is limited evidence for the effectiveness of keyboards with an alternative geometry, and limited evidence for breaks during computer work compared to no breaks. There is need for better targeted, higher quality research.*
Boocock, 2007	Aim: To evaluate findings of primary/secondary and/or tertiary intervention studies for neck/upper extremity musculoskeletal conditions undertaken between 1999 and 2004.
	*Conclusion: Some evidence for work environment/workstation adjustment for improved health outcomes in VDU workers with neck/upper extremity conditions.*
Kennedy, 2009	Aim: To answer the question: “do OHS interventions have an effect on upper extremity musculoskeletal symptoms, signs, injuries, claims and lost time?”
	*Conclusion: We recommend that worksites not engage in OHS activities that include only workstation adjustments. However, when combined with ergonomics training, there is limited evidence that workstation adjustments are beneficial. A practice to consider is using arm supports to reduce upper extremity MSDs.*
Leyschon, 2010	Aim: to determine the level and quality of evidence supporting ergonomic interventions to improve the comfort, safety and/or productivity of office workers with symptoms of MSDs
	.*Conclusion: There is still limited quality research that addresses ergonomic interventions designed for secondary prevention. Further high quality studies are needed to support evidence-based ergonomic interventions in practice. For all stakeholders to fully evaluate the usefulness of the ergonomic intervention studies need to attend to outcomes not only of worker comfort but also to productivity and safety.*
Driessen, 2010	Aim: to investigate the effectiveness of ergonomic interventions (physical and organizational) in reducing the incidence/prevalence and intensity of LBP and neck pain among non-sick listed workers.
	*Conclusion: Ergonomic interventions that combined the use of an arm board support and ergonomic training were significantly more effective in reducing neck pain intensity than ergonomic training only. As regards the use of a trackball, no significant effects were reported on neck pain intensity. Based on the significant reduction in neck pain intensity found in this single study, there is low quality evidence that a physical ergonomic intervention (i.e., arm board support) is significantly more effective in reducing neck pain intensity in the long term.*

## Discussion

### Summary of main results

A critical, systematic overview of the evidence for the overall causal relationship between computer work and development of CTS and UEMSDs revealed that there is insufficient evidence for a causal relationship between computer work and CTS, and that the relationship between computer work and UEMSDs shows a more mixed level of evidence. Reviews on UEMSDs have become more cautious since the statements in 1999 [10], [11], apparently coinciding with the availability of more prospective studies and the use of more rigorous outcome definitions. The reviews indicate an association between pain complaints and the intensity of computer use, but do not support evidence for an association between aspects of computer use and specific disorders of the neck and upper extremity. Interestingly, recent studies even challenge the reported association between pain – at least chronically and prolonged – and computer work. In two cohort studies using objective measures of computer use and not included in the reviews, no association could be established between measures of more prolonged and chronic pain and computer use [55]–[57], (Mikkelsen S, personal communication].

### Completeness and applicability of evidence

#### Completeness of evidence

The reviews included are sufficient to address the objectives of the overview. The three reviews on CTS were consistent in their agreement on an insufficient evidence for computer use leading to CTS. As all reviews were considered to be of medium to high quality the conclusions about causation can be considered fairly certain.

The reviews on UEMSDs were far less specific than the CTS reviews and not all combinations of computer use and types of outcome have been studied in detail. In the majority of reviews, outcomes from the neck and upper extremities were described as “pain complaints”, defined in various ways. There was some evidence across reviews for an association between computer use and pain complaints, and this relation could be causal, but the argument for a causal relationship would be more convincing if there had been a demonstrable threshold. The review which specifically included diagnoses [32] found limited evidence for a causal relation between computer use/mouse use and tension neck syndrome and wrist tendonitis and for mouse use and forearm disorders. However, the conclusion concerning wrist tendonitis was partly based on [36], [54], and it should be noted that the original papers refrained from such a conclusion due to a very small number of cases despite a large cohort. Waersted et al. [32] further found insufficient evidence for shoulder tendonitis and epicondylitis [lateral or medial]. There was no evidence across reviews for a causal relation between computer use and specific disorders of the neck and upper extremities. The evidence across reviews of intervention studies was fairly consistent in showing limited evidence for the effect of specific interventions. At best a multi-targeted strategy could be recommended, if there was a call for an intervention.

#### Applicability in the context of current practice

The aim of this overview was to assess the current evidence for possible causal relationships between computer work and CTS and UEMSDs, and to synthesize possible recommendations for intervention in the work place. The reviews of risk factors did not give sufficient information to derive quantitative dose-response relationships, and the findings from the studies that indicated a relationship between duration of computer use and pain complaints could not indicate a threshold value. Without such quantifications we cannot derive the duration and/or the degree/load/force involved in the work situation that will result in an increase in the occurrence of UEMSDs and CTS. A prerequisite for the combination of information in different studies would be that the different studies had used sufficiently similar measures of exposures and outcomes.

### Quality of the evidence

The quality of the included reviews was assessed by AMSTAR, while the quality of evidence for the overall key areas of interest across reviews was assessed using a qualitative approach taking into account the consistency of the reviews, the overall likelihood that the observed associations were due to a causal relationship, and the capability of intervention studies to provide evidence for practical and effective work place recommendations.

#### Quality of the included reviews

All included reviews – except [14], [22] - reported at least some details about their literature search, selection process and quality assessment. The lack of methodological rigorousness in the two review [14,22] was reflected in the low AMSTAR score obtained by these two reviews compared with the others.

The scopes of the reviews were different. The three reviews on CTS focused on one specific outcome, and generally obtained higher quality scores than the reviews looking more broadly on UEMSDs. The study aim was rather diffuse and general in two reviews [14], [22], and again reviews with more precise aims obtained a higher quality score. The tendency for some reviews to rely exclusively on cohort studies is questionable. In studies of musculoskeletal outcomes, cross-sectional studies and other study types than cohort studies may indeed provide valuable information. The two reviews by Gerr et al. acknowledge this [23], [24], and maybe this is not fully rewarded by the AMSTAR scores. The most recent review on risk factors on the other hand included intervention studies in their appraisal of evidence for causal relationships, but this seems to be a questionable approach as long as risk factors or risk agents intervened against are not well established.

It is noteworthy that all the reviews of intervention studies scored relatively high on the AMSTAR criteria, but that may actually cast some doubt about the appropriateness for using AMSTAR in public health studies. In our opinion two of the intervention reviews [34], [35], which originated from the same group, had a higher quality than the others, based on their completeness, thorough search strategies, and rigorousness of interpretation, but this was not fully reflected in the AMSTAR scoring.

#### Quality of evidence for topics of key interest

The quality of the evidence regarding computer use and CTS was considered high. The three reviews were consistent in their conclusions and the results seem well founded. For UEMSDs the reported evidence ranged from insufficient over limited to consistent and well established, and the quality of the evidence was considered moderate. It cannot be excluded that future studies will change or modify the conclusions on UEMSDs. The reviews of interventions were consistent in their conclusions on the lack of high quality studies and the shortage of evidence based recommendations applicable in today's office environment. The quality of the evidence for this statement was considered high and there is no reason to believe that important information from intervention studies has been missed.

### Potential biases in the overview process

#### Potential biases related to inclusion of reviews

Authors of all the included reviews were contacted to ensure that any substantial reviews or ongoing projects was not overlooked, and we are not aware of any research group currently performing comprehensive reviews or meta-analysis. Five reviews were excluded due to language. However, based on the English abstracts included in the reviews and their reference lists we do not believe substantial information was missed. The whole area of grey literature was not formally searched, but we screened some reports for studies, which were not accounted for in the included reviews. The 2001 consensus report from National Research Council [58] included 9 of the 80 papers on risk factors covered by the six reviews in this paper. A Norwegian report on work as causal for musculoskeletal disorders from 2008 obtained information from 7of the risk factor studies, 11 from the intervention studies, and they included 6 of our 17 reviews [59], and an European report from 2008 [60] extracted their information from 2 of the reviews in this paper [26], [31]. From these three examples it can be concluded that commissioned reports attempting to cover exposures and outcomes very broadly, ends up basing their evidence on a smaller number of original studies than most of the reviews in this paper.

#### Potential biases related to conceptualization

It has been shown that the single largest source of error in relation to sensitivity in searching is errors in conceptualization of the search [61]. The very first step in evidence based medicine is to formulate questions that can be answered [62]. If nothing else, it became clear that the included reviews with the lowest AMSTAR score also asked the most imprecise questions.

#### Potential biases related to searching and study selection

Our search strategy involved a librarian with many years of professional experience, and we searched both in Medline, Embase, CINAHL and Web of Science. A complementary search in the Cochrane database did not reveal additional reviews. We consider our search strategy to cover the important information for our research questions.

There was a poor overlap between the studies selected in the reviews on risk factors of UEMSDs and intervention studies, whereas the overlap for assessing risk factors for CTS was much higher. Probably the bias in this overview is lower than in most of the included reviews, because the information from all the reviews was taken into account.

#### Potential biases related to analysis and synthesis

The AMSTAR scoring was performed independently by two authors and there were very few disagreements, which all could be solved by discussion. In the synthesis of the overall evidence we made a qualitative assessment rather than a formal one. This could be criticized, but we find measures such as GRADE developed to suit public health studies less well than studies on pharmaceutical interventions. A formal system to synthesize the evidence into conclusions would have been preferable, but to our knowledge nobody has set up such a system and tried it out in relation to reviews of etiology or public health interventions.

### Agreements and disagreements with other studies or reviews

#### Agreements and disagreements with other overviews

We could not identify other overviews with the same aim as our own.

#### Agreements and disagreements with previous views or seminal reviews

The research conducted in the last decade has clearly led to more modest conclusions on the relation between computer work and the risk of CTS and UEMSDs, and demonstrated the lack of well documented recommendations concerning interventions in the office environment. This development is a cause for concern. If there were a causal relation between computer work and CTS and UEMSDs, it would have been expected that the introduction of better study designs, larger studies, and more rigorousness in analysis, had strengthened the evidence, but apparently the opposite has been the case. The explanation for this is not straight forward. It's seems unlikely that changes in the office environment over the last 10 to 15 years should have reduced previous exposure levels and made computer related MSDs a thing of the past. It may be argued that office ergonomics and work postures have been improved, but at the same time we have witnessed work intensification and an increased mouse use.

An alternative explanation would be that the alarming reports from earlier studies reflected a public anxiety of new technology, more medicalization of society and the radical change of work organization in the office environment rather than a traditional association between high physical work place exposure and adverse health outcomes. Perception of pain and subjective feelings of discomfort in association with computer work is not a trivial issue, whether the association is causal or not. The effects in terms of loss of productivity, reduced well-being at work and a negative influence on psychosocial work conditions are substantial and should be of major concern for everyone involved in working environment issues and occupational health. The solutions, however, appear to be complex, involving a system approach, focusing on safety management systems and work organizational changes based on participation and dialogue. In this perspective, equating pain and discomfort from computer work with musculoskeletal injuries in traditional industrial work seems to be an unfruitful approach.

### Authors' conclusions

There is moderate to high quality evidence indicating an increased risk of acute or transient pain complaints among computer users, when they are keying or using their mouse intensively, but a causal relation is still uncertain. There is no evidence for specific diseases or chronic pain development. There are no effects of preventive interventions that include only workstation adjustments. There is limited evidence that a combination of ergonomics training with workstation adjustments may be beneficial.

### Implications for practice

The available epidemiological literature does not present sufficient evidence to derive that computer work could lead to clinically relevant increases in the occurrence of CTS or UEMSDs. There are no strong recommendations for specific interventions at the work place, pointing to a combination of strategies as the most reasonable approach.

### Implications for research

Improved exposure assessment methods using the available technology for direct measurements in combination with more rigorous outcome definitions would improve future epidemiological studies in the area. It should, however, be emphasized that in the light of the lack of evidence of the occurrence of clinical outcomes related to computer work, such conditions, if they exist, are deemed to be seldom, and it may be the time to weight the effort involved into creating huge cohorts in the office environments against the benefits that can be gained from research related to other occupational health concerns.
